# Exercise behaviors and barriers to exercise in adult burn survivors: A questionnaire survey

**DOI:** 10.4103/2321-3868.123075

**Published:** 2013-12-18

**Authors:** Jennifer Baldwin, Frank Li

**Affiliations:** 1 Physiotherapy Department, Concord Hospital, Hospital Road, Concord, Sydney, New South Wales Australia; 2 Physiotherapy Department, Concord Hospital, Concord, Building 30, Sydney, New South Wales 2139 Australia

**Keywords:** Burns, exercise behaviors, survey

## Abstract

Exercise is a key component of burn rehabilitation across all phases of care. Supervised outpatient exercise programs have been shown to improve outcomes following burn injury. However, little is known about the exercise behaviors of burn survivors who do not undertake such programs. This study aimed to investigate self-reported exercise behaviors and barriers to exercise in adult burn survivors. A short questionnaire survey was conducted on adult burn survivors attending the ambulatory burns clinic of a tertiary hospital over a 6-week period. The collected data were subjected to paired *t*-tests and Pearson’s correlation test. A total of 63 adult burn survivors (mean age 36.5 years) completed the questionnaire. Participants reported exercising less frequently and engaged in fewer different types of exercise compared with pre-burn (*P* < 0.05). Poor physical conditions and low motivation and enthusiasm were the major barriers to exercise. Participation in supervised exercise programs can be limited by a range of factors including the availability of resources and access to facilities. This preliminary study suggests that there is a need to improve compliance with outpatient exercise programs. Burn survivors appear to exercise less frequently after burn injury. Barriers to exercise following burn injury include poor physical condition and reduced motivation. Further investigation into overall physical activity following burn injury and potential physical and psychological limitations is warranted. Burn clinicians should highly encourage injury survivors to participate in supervised exercise programs when available or to do exercises at home to maximize post-burn injury recovery.

## Introduction

As burn injury-associated mortality rate has been consistently decreasing over recent decades,[1] reducing burn-related morbidity constitutes an increasingly significant challenge to burn care clinicians and researchers. The process of recovery following burn injury is often lengthy and complex. Common sequelae include scarring, wound breakdown, pain, contractures, joint stiffness, and physical deconditioning. Physical deconditioning is characterized by reductions in lean body mass and aerobic capacity and is associated with deleterious effects on wound healing, infection risk, morbidity, and physical function.[2] The two major causes of physical deconditioning following severe burn injury are muscle catabolism and bed rest.[3] Physical condition and function are important outcome measures following burn injury, and it has been previously demonstrated that the functional independence measure (FIM) score for locomotion is the single most important determinant of discharge destination.[4]

**Table:** 

Access this article online	
**Quick Response Code:**	**Website:** www.burnstrauma.com
	**DOI:** 10.4103/2321-3868.123075

Exercise is essential to improving strength, aerobic capacity, and overall function in burn survivors across all phases of recovery.[5] Exercise following burn injury has been previously shown to be safe and to have no effect on the hypermetabolic response.[6] Supervised exercise programs have demonstrated improvements in overall physical function,[7,8] lean body mass,[6,9,10] strength,[6–11] aerobic capacity,[3] exercise tolerance,[7,8] pulmonary function,[8,12] and quality of life,[8] as well as reduce the need for surgical interventions.[13] Such exercise programs have typically consisted of a combination of strength and aerobic training in addition to stretching exercises for three sessions per week. [7,8,11–13]

Geographical isolation and limited funding affect access to tertiary burn care and rehabilitation centers, particularly in Australia,[8] rendering participation in supervised exercise programs difficult for many burn survivors. At present, little is known about the exercise behaviors of burn survivors who do not undertake supervised exercise programs. The term “exercise” denotes physical activity for the purpose of health or fitness, and “behavior” refers to the behavioral patterns related to a particular act.[14] Hence, the term “exercise behaviors” describes the behavioral patterns relating to physical activity undertaken for fitness. Given the role of exercise in improving outcomes following burn injury, the evaluation of exercise behaviors and barriers to exercise is warranted. The aims of this study were: (1) To investigate self-reported exercise behaviors in adult burn survivors and comparing participation in exercise prior to and after injury and (2) to identify perceived barriers to exercise following burn injury.

## Materials and Methods

### Participants

Adult burn survivors who attended the Ambulatory Burns Clinic at the Concord Hospital, Sydney, Australia during the 6 week’s study period were considered potential participants. Potential participants presented to the clinic at varying time points in their recovery. They had been either admitted or treated as outpatients, and the treatment involved either surgery or conservative management. Individuals with insufficient English skills or a cognitive or behavioral deficit affecting their ability to complete a written questionnaire were excluded. Consent was obtained from all participants. The research project was approved by the Ethics Department of the authors’ hospital.

### Questionnaire

A simple questionnaire survey, prepared in English, was conducted. The questionnaire consisted of questions pertaining to the participant’s demographic details, burn injury and subsequent management, and exercise behaviors and barriers prior to and following burn injury [Appendix 1]. Details regarding burn injury and management were validated via chart review.

Responses to questions 6 and 9 on the questionnaire regarding exercise frequency are quantified on an ordinal scale as follows:

- 1 = Less than once per week
- 2 = Once per week
- 3 = Several times per week
- 4 = Most days per week
- 5 = Every day

## Statistical analysis

The statistical software Statistical Package for Social Sciences (SPSS; Chicago, IL, USA) was used. The data were analyzed using paired *t*-test and correlation coefficients. Differences were considered significant when *P* < 0.05.

## Results

### Demographic information

In total, 69 adult burn survivors (52 male) completed the questionnaire. Six participants reported experiencing their injury greater than 3 years ago; data from these participants were not included due to likely error from patient recall. Demographics and burn injury history for the 63 participants included in the data analysis are detailed in Table 1.

**Table 1:** Demographics of participants with a burn injury in the past 3 years included in the study

	Mean (SD)	Range
Age (years)	36.5 (13.9)	17–73
Burn TBSA (%)	14.9 (18.3)	1–89
Time elapsed since injury (months)	5.7 (7.3)	0.5–27.0
Average hospital LOS (days)	21.3 (42.7)	0–270

SD = Standard deviation, TBSA = total body surface area, LOS = length of stay

### Burns in different parts of the body change the exercise behaviors of patients

**Figure 1:**
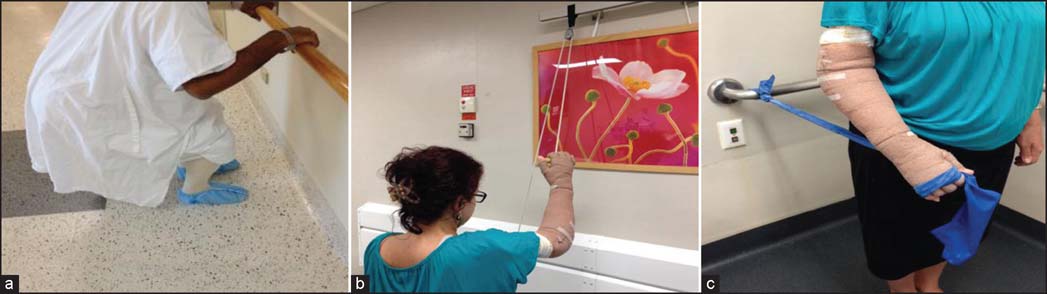
Exercises patients performed post-burn. (a) Active exercises for stretching and strengthening. (b) Active assisted exercises using pulleys. (c) Strengthening exercises with theraband.

**Figure 2:**
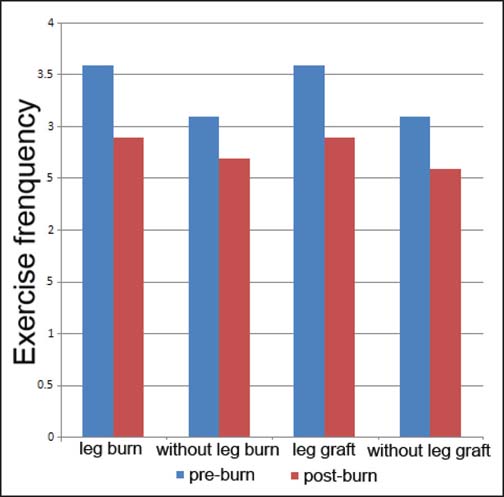
Self-reported exercise behaviors pre- and post-burn in individuals with or without leg burns and skin grafting to legs. No significant differences were found.

**Figure 3:**
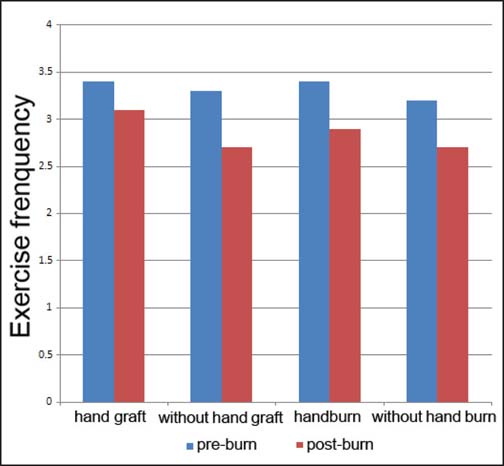
Self-reported exercise behaviors pre- and post-burn in individuals with or without hand burns and skin grafting to hands. No significant differences were found.

The reported changes in exercise behaviors from pre- to post-burn injury are summarized in Table 2. Participants reported exercising less frequently following their burn injury compared with prior to their injury (*P* = 0.02). Participants also engaged in fewer different types of exercise (*P* = 0.001) and performed strengthening exercise less frequently following their injury (*P* = 0.018) [Figure 1]. There was no change in stretching or aerobic exercise frequency. When exercise frequency was analyzed in relation to the presence or absence of leg or hand burns, all groups demonstrated a reduction in exercise frequency; however, there was no significant difference in the size of this reduction between groups [Figures 2 and 3]. No correlation was found between post-burn exercise frequency and age, hospital length of stay (LOS) or total body surface area (TBSA) burned.

**Table 2:** Changes in exercise behaviors post-burn

	Pre-burn mean (SD)	Post-burn mean (SD)	Size of effect (*P*-value)
Exercise frequency per week*	3.3 (1.41)	2.8 (1.3)	0.5 (0.02)
Number of different types of exercise	2.1 (1.5)	1.6 (1.1)	0.5 (0.001)
Number of barriers to exercise	1.0 (0.8)	1.4 (1.2)	0.4 (0.001)
Barriers to exercise	Time	Physical condition Motivation	N/A (0.001)

*Exercise frequency quantifed on a 5-point scale where 1 = less than once per week and 5 = every day

### Barriers to exercise after burns

Participants experienced a greater number of barriers to exercise after their burn injury (*P*= 0.001) [Table 2]. Poor physical condition (*P* = 0.000) and low motivation (*P* = 0.011) were identified as barriers to exercise following burn injury, whereas time was a barrier prior to injury (*P* = 0.001).

## Discussion

This study was a preliminary investigation into exercise behaviors in adult burn survivors. Current American College of Sports Medicine guidelines for exercise recommend that individuals undertake moderate physical activity for 30 min on most days of the week to obtain health benefits.[15] In burn rehabilitation, guidelines for outpatient exercise programs are based on previous literature, advocating a combination of strength and aerobic exercise on three occasions per week in addition to daily stretching exercises and regular activities.[3,7–9,11–13] In this study, participants reported an exercise frequency of less than several times per week following their injury, which is below that of the recommendations for both healthy individuals and burn survivors. Burn survivors in this study therefore may not be engaging in sufficient exercise to gain the known benefits.

In this study, the severity of the initial injury was not correlated with exercise behaviors. No correlation was found between burn TBSA or hospital LOS and exercise frequency, and the presence of hand or leg burns had no impact on the change in exercise behaviors. However, poor physical condition was found to be a significant barrier to exercise following burn injury. It appears that perceived physical condition may be influenced by physical or psychological factors other than the initial injury, although information regarding actual physical condition was not collected. It is possible that comorbidities, pain, social supports, prior life experience, and previous physical condition may influence the individual’s perception of their condition. Physical data were not collected in this study and are a direction for future research investigating the impact of physical and psychological factors on exercise performance.

The outpatient exercise protocol at the authors’ institution consists of exercise prescription for individualized, home-based exercise programs. Active range of motion is emphasized as this has been identified as the best exercise for joints affected by burns.[16] Upon discharge from the hospital, individuals are given education regarding the importance of exercise following burn injury and are prescribed a home exercise program in which they practice with the therapist prior to discharge. Home exercise programs comprise daily active range of motion and stretching exercises, as well as three sessions each of aerobic exercise and strength training per week. The type of exercise prescribed varies depending on individual needs, but examples of common aerobic exercise include walking, cycling, and jogging. Individuals living locally are followed-up at the hospital clinic once to three times per week for therapist-assisted range of motion exercises and to progress their home program. Individuals living out of Sydney are referred to their local physiotherapist or exercise professional. Due to limited resources, outpatients do not undertake supervised strengthening or aerobic exercise at the hospital clinic. From the results of this study, burn survivors may not have received adequate exercise prescription and education upon discharge from hospital or may not have been adequately followed-up in the outpatient setting. The role of health professionals in prescribing, monitoring, and progressing individual exercise programs, particularly when supervised exercise is not undertaken, is emphasized.

There are several limitations to this study. The questionnaire used was developed for a quality improvement project and has not been evaluated for validity or reliability. There are several shortcomings within the questionnaire, for example the reliance on the recall of exercise behaviors prior to and following burn injury, as well as the use of self-reported information. The assessment of physical activity by self-report has been shown to overestimate actual exercise levels,[17,18] and the recall of exercise behaviors prior to burn injury in cases of greater than several elapsed years is questionable. For this reason, only those participants injured within the past 3 years were included in the data analysis. Additionally, no data were collected regarding the duration or intensity of exercise; these are important to consider when assessing exercise behaviors. The different types of exercise were not clearly defined, possibly resulting in exercise misreporting. For example, the term “stretching exercise” was intended to incorporate stretches and range of motion exercises; however, this may not have been apparent to the participant. Furthermore, the questionnaire examined formal exercise only and did not assess other components of physical activity such as incidental, occupational, domestic, or transport-related physical activity. There are several standardized questionnaires available for assessing self-reported overall physical activity, for example the International Physical Activity Questionnaire[19] or the Seven-Day Physical Activity Recall.[20] Finally, many factors potentially influencing participation in exercise following burn injury were not investigated, for example pre-morbid physical or psychological conditions and the physical and psychological changes associated with burn injury. These factors are important considerations for both clinicians and researchers. Findings from this preliminary study highlight the importance of initiating and monitoring individual exercise programs and inform possible future directions for research.

## Conclusion

This preliminary study found that adult burn survivors exercised less frequently and engaged in fewer types of exercise following their burn. The exercise frequency reported by participants was below that of current recommendations for both healthy populations and burn rehabilitation. Participants experienced a greater number of barriers to exercise post-burn and reported physical condition and motivation as significant barriers. There was no correlation between exercise frequency and age, burn TBSA, or hospital LOS. Further research to investigate overall physical activity using a validated questionnaire and the impact of pre-morbid and current physical or psychological conditions on participation in exercise is indicated.

### Clinical implications

The treating therapist plays a key role in prescribing and monitoring exercise programs for adult burn survivors, particularly in situations where participation in structured exercise programs may not be feasible. Physical and psychological factors potentially affecting participation in exercise are important considerations for therapists.

## Appendix 1

Burn Injury and Exercise Questionnaire Date:

Please circle the corresponding answer/s (there may be more than one), and provide specific details where required.

Your age:__ Gender: Male Female

1. What was the date of your burn injury? __
2. What areas of your body were burned? Feet Leg/s Buttocks Trunk Arm/s Hand/s Neck Face
3. What percentage of your body was burned? __
4. Did you require surgery for skin grafting? Yes No if yes, please specify what area(s) were grafted. __
5. How long was your hospital stay?__ Months__ Weeks __ Days Not hospitalized
6. What was your employment status prior to your burn injury? Employed: Full-time Part-time Please specify your occupation: __ Not employed Student Retired Pensioner
7. What is your current employment status? Employed: Full-time Part-time Please specify your occupation: __ Not employed Student Retired Pensioner

The following questions relate to exercise. Please answer the questions as best you can, keeping in mind how you feel on most days of the week.

8. Prior to your burn injury, how often did you exercise? Less than once per week Once per week Several times per week Most days per week Every day
9. Please list the type(s) of exercise you took part in prior to your burn injury. Walking Swimming Cycling Weights training Stretching Other (please specify)__
10. Prior to your burn injury, what factors affected your ability to exercise on a routine basis? Time Motivation Physical condition Access to facilities Cost Other (please specify)__
11. Since your burn injury, how often do you exercise? Less than once per week Once per week Several times per week Most days per week Every day
12. Please list the type(s) of exercise you take part in regularly since your burn injury. Walking Swimming Cycling Weights training Stretching Other (please specify)__
13. How often do you exercise now compared to before your burn injury? Significantly more often Slightly more often About the same Slightly less often Significantly less often
14. Since your burn injury, what factors now affect your ability to exercise on a routine basis? Time Motivation Physical condition Access to facilities Cost Physical appearance Motivation Feeling self-conscious Other (please specify)__
15. Has your burn injury affected your motivation to exercise? More motivated No change Less motivated Unsure
16. Has your burn injury changed the type of exercise you do? Yes No Unsure Don’t exercise regularly Thank you for taking the time to complete this questionnaire.
